# Psychometric properties of the of the Persian version of the Coping Assessment for Bereavement and Loss Experiences (CABLE)

**DOI:** 10.1186/s12888-022-04338-8

**Published:** 2022-11-19

**Authors:** Abbas Ebadi, Asal Seraji, Maryam Farmahini Farahani, Hamid Sharif Nia

**Affiliations:** 1 Behavioral Sciences Research Center, Life style institute, Baqiyatallah University of Medical Sciences, Tehran, IR Iran; 2 Nursing Faculty, Baqiyatallah University of Medical Sciences, Tehran, IR Iran; 3 Department of Nursing, Faculty of Nursing and Midwifery, Tehran Medical Sciences, Islamic Azad University, Tehran, Iran; 4 Department of Midwifery, School of Nursing and Midwifery, Tehran Branch of Medical Sciences, Islamic Azad University, Tehran, Iran; 5 Psychiatry and Behavioral Sciences Research Center, Addiction Institute, Mazandaran University of Medical Sciences, Sari, Iran

**Keywords:** Coping, Grief, Psychometrics, Persian

## Abstract

**Background and aim:**

The loss of a loved one through death is practically an inevitable part of the human experience. However, not all grieving people cope with this blow in the same way. One of the factors that may differentiate the grieving reactions of mourners in the face of this lesion is the strategies that the person uses in this situation to adapt and manage the situation. A valid and reliable tool is also needed to measure and evaluate coping strategies. The aim of this study was to translate and determine the characteristics of psychological tools for measuring coping with experiences of grief and loss (28 items) in people living in Tehran.

**Materials and methods:**

This is a methodological study with a descriptive cross-sectional design that after obtaining written permission from the original developer and according to the WHO protocol, the Persian version of the questionnaire was completed by 480 people who experienced mourn in Tehran in February 2021 to October 2021. Then, the Face validity, Content validity and Construct validity of questionnaire were assessed. Cronbach’s alpha coefficient, McDonald’s omega and Test-retest were used to determine the reliability.

**Results:**

Cronbach’s alpha for all items was 0.91 and intra-class correlation coefficient was 0.86, both of which indicate the reliability of the Persian version of the CABLE tool. Based on exploratory factor analysis, maximum likelihood (*n* = 260) and confirmatory factor analysis (*n* = 220) six factors were identified. Factors can explain 50% of the total variance observed. The model had an acceptable fit: GFI: 0.88, CFI: 0.96, IFI: 0.96, NFI: 0.92, PNFI: 0.82, RAMSEA: 0.058, CMIN / DF: 2.37 RMR: 0.056. Internal consistency and construct validity of the questionnaire were confirmed.

**Conclusion:**

The findings of the present study indicate that the Persian version of CABLE has the appropriate validity and reliability to assess the compliance with the experiences of grief and loss in Persian population.

## Introduction

Mourning is an unavoidable phenomenon [1, 2] in all people around the world that each person experiences it differently and this phenomenon can affect all aspects of a person’s life [3, 4]. The psychological feeling caused by the experience of the death of a loved one is called mourning [2, 5, 6] Also in Dehkhoda dictionary, mourning has been interpreted and equated with the concept of misery, grief and mourning (following the death and loss of loved ones) [7]. The turning point and common point of all texts related to mourning is the experience of this phenomenon following the lack [8]. Man, who is a social being and has the ability to establish strong emotional bonds, experiences this phenomenon (mourning) after breaking these bonds or losing them following death [9]. .All groups, from the smallest unit, which is the family, to the whole community, have a normative framework called culture, which is dominated by the behaviors of individuals and how rituals are performed and how they are performed (culture). Inspired [10]. One of these behavioral acts is grief. We all belong to social groups that have internalized the prevailing norms for how to grieve [11]. On the other hand, in many studies, religion and spiritual approach are considered important and effective in how to deal with the experience of loss and mourning and the way of mourning [12, 13]. The prevalence of grief and its consequences vary due to differences in social norms and cultural expectations [3, 14]. Studying the concept of mourning is difficult and complex, not only because of individual and cultural differences, but also because of the different prevalence in different societies [15, 16]. In the meantime, mourning is critical due to its multiple nature, followed by loss [17, 18]. .As a global crisis, with the outbreak in the first half of 2020, Covid 19 drastically changed human relations, norms, and the quality of experiences of loss and grief at the population level [19]. It is predicted that societies will face very important and dangerous periods in this crisis due to depression, anxiety and post-traumatic stress caused by grief and the experience of loss [4, 19]. In most cases, the period of mourning is naturally accepted and spent by the bereaved as a fact of life [20], but not all people adapt in a style to the experience of loss, and among these, 10–15% of the bereaved and mourning population show an incapacitated and life-threatening [21]. One of the factors that may differentiate people’s reactions to grief is how they adapt and coping to the experience of absence [22]. Identifying adaptive coping strategies and practices is very important and necessary for mental health professionals to intervene to support the grief and injury caused by bereavement and loss [23]. However, one of the barriers to identifying and evaluating grief coping strategies and the experience of loss is the lack of specific tools related to the concept of grief to evaluate the potential constructive coping strategies for grieving. In 2017, Crank et al. Developed a proprietary tool for coping with grief and the experience of loss, and is currently the only CABLE 28 proprietary tool. The CABLE tool is a dedicated benchmark for evaluating and identifying coping and coping strategies that is quite cost-effective and can be used by both researchers and therapists as well as by individuals who can respond. Therefore, in this study, the ability to use special CABLE tools to assess coping with grief and the experience of loss in Iranian society and culture, with the aim of translating and determining the characteristics of psychometrics, was examined.

## Methods

### Ethical considerations

The objectives of the study were explained to the participants. All of them were provided with the necessary explanations in the attachment sheet of the virtual questionnaire regarding the confidentiality of their information with the researchers. This study was conducted in accordance with the Helsinki Declaration.

### Participants

The sample of this study includes all people over 18 years of age with a history of grief experience that is at least 6 months old. The questionnaire was made available to individuals virtually through social networks. The issue of sample size adequacy is considered an important and serious issue in methodological studies, but there is no definite idea about the appropriate sample size and various instructions have been provided. One of these guidelines is the rule of thumb, which suggests at least 300 examples for such studies. On the other hand, based on the number of questions in the study tool, 10 participants per question were considered appropriate. In this study, due to having 28 items, 280–300 participants are appropriate [24]. The questionnaire was provided to the participants electronically and in person, and finally 480 questionnaires were completed and delivered.

### The coping assessment for bereavement and loss experiences (CABLE)

CABLE is a scale designed to identify coping strategies that mourners use to cope with Bereavement and loss. This scale consists of 28 items that are followed by convergent validity in the original version of CABLE with the Brief Cope scale. And is set in 6 areas: Help Seeking (7 items), Positive Outlook (5 items), Spiritual Support (4 items), Continuing Bound (5 items), Compassion Outreach (3 items), Social Support (4 items) and its response On a scale of 6 to zero (I have never done this), (I have done this once), (I have done this many times), (I do it almost daily I have done this, (I have done this every day), (this does not apply to my menu and my mourning). Cronbach’s alpha coefficient of the total scale was 0.91 [23, 25].

### Study design

The present study is a methodological research that has been conducted with the aim of translating and psychometric evaluation of the Persian version of the questionnaire to assess compliance with grief and the experience of loss from April 2020 to April 2021 in the form of a cross-sectional descriptive design.

### Validity and reliability

The translation is performed according to the standard defined in the validity of the instrument by the World Health Organization. After obtaining written permission from the original designer of the “CABLE” tool, using the World Health Organization guide and the Forward method, the Validity and Reliability began [26, 27]. In the first stage, the “CABLE” questionnaire was translated into Persian by three translators whose mother tongue was Persian and who had sufficient experience and proficiency in translating English texts. During the translation, an attempt was made not to change the meaning and concept of the phrases and their level of difficulty. In this regard, in the Validity and Reliability, the conceptual equivalence of words, sentences and phrases was emphasized. In the second stage, the original translated versions were reviewed and compared by experts, and the discrepancies between them were corrected and the original translations were merged. Then, in the third stage, the independently translated version was translated into English by two fluent English speakers (a Canadian citizen doctor and a skilled English translator), and then the agreed version was reviewed. The final prepared version was sent to the original designer for approval to compare with the original version. After submitting his comments and suggestions, the final written approval was received.

### Face and content validity

Cognitive interviews were used to perform face validity. The Persian version of the scale was provided to 15 people with experience of mourning, over 18 years of age with a minimum level of literacy, and qualitative face validity was performed. Due to the existing cultural-socio-religious differences, limited changes were made in some words. Then in reviewing the content validity by qualitative method, 10 experts (respected university professors with experience in methodology, psychometrics, instrumentation, psychology and nursing) in this field were asked to, after careful study of the instrument, their corrective views on observe the grammar, use the right words, put the items in the right place and grade the scale in writing [28, 29].

### Item analysis

The item of analysis has been done with the aim of checking the initial reliability, examining the effect of each item on the amount of reliability and identifying problematic, incorrect items and correcting them. At this stage, the final and modified version of the scale was given to 30 participants. Using SPSS 26 software and loop technique, the correlation between items and the correlation of each item with the total score were measured. Cronbach’s alpha was also described after removing each item.

### Construct validity

In the CABLE psychometric process, the issue is cultural compatibility and the concept is intercultural. We also had slight changes in the content validity. Based on this, the opinion of the professors and the research team was to do EFA first and then CFA**.** Exploratory factor analysis (*n* = 260) was used to determine the Construct validity. In this method, Kaiser-Meyer-Olkin test (KMO) was used to evaluate the adequacy of sampling. (KMO > 0.7) Measurement of KMO close to 1 indicates developmental adequacy. High negation in factor analysis and KMO between 0.7–0.8 and above 0.8 is considered good. Then the correlation matrix between the variables was evaluated using Bartlett test for factor analysis with an error level of less than 0.05. To extract the factors, the maximum likelihood (ML) and Scree design were used. Also, the rotation of factors in this study was done by rotation of Promax. Data analysis in exploratory factor analysis (EFA) was performed using SPSS27.

**Fig. 1:**
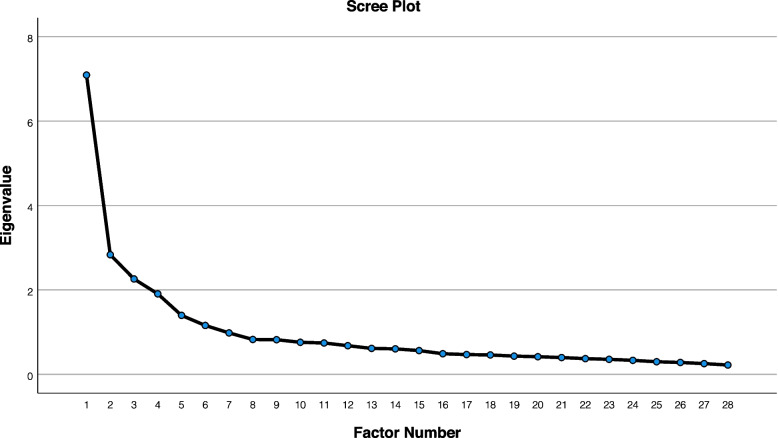
Scree Plot

For confirmatory factor analysis was done on different samples include 220 individuals were surveyed according to inclusion criteria and by sampling method. In confirmatory factor analysis, several change techniques were used to evaluate the relationships. In order to fit the model, chi-square, chi-square to degrees of degree of error scales, goodness-fit index, standardized fit index, and adaptive fit index are used, which are commonly used in determining fit in factor analysis. In examining the goodness indicators of fit, if the result of the chi-square test is not statistically significant, the fit of the model indicates. The first degree error criteria show the fit for each of the freedoms, and the closer the model is to the greater zero of the model. If the square root of the error is less than 0.08, it indicates a very good fit, 0.08 to 0.1 indicates an acceptable fit, and greater than 0.1 indicates a poor fit of the model. Goodness indicators of fit, handled fit and adaptive fit are indicators that have been developed to compare the model and are calculated with a base model. Their online is between zero and 1 and above 0.9 indicates a good fit of the model [30]. Data analysis in CFA was performed using AMOS 27. We also examined the scree plot to cross validation factor selection. (Fig. 1).

### Normality and missing data

Univariate distributions were investigated for outliers, skewness, and kurtosis through evaluating skewness (±3) and kurtosis (±7) and Multivariate outliers were evaluated through the Mahalanobis squared distance (*P* < 0.001). The distribution of missing data was also assessed using multiple imputation and then, missing data were changed by the mean score of participants’ responses.

### Reliability

Reliability was assessed by two methods of internal consistency including Cronbach’s alpha and McDonald’s omega and stability with test-retest. In the open test phase, the scale was given to 30 subjects in two stages 2 weeks apart. Then the intraclass correlation coefficient (ICC) was calculated with a confidence interval of 0.95 and values above 0.7 were accepted for scale stability.

## Results

### Samples

The mean age of participants in this study was 18–86 years, of which 56.6% were women. 15.3% of them in the last 6 months, 17.3% in 6 months to 1 year, 29.4% in 1–5 years and 38% of them have more than 5 years of experience of grief and loss.

### Face and content validity

After review by respected faculty members and experts, some cases were changed and adjusted due to religious-cultural differences. For example, in question 13, instead of the Bible and prayer, the Qur’an, texts and prayers were used, and in question 16, in addition to the synagogue and the church as religious places, the mosque was also mentioned. In other cases, there was no cultural difference and simpler and similar expressions were used. In content validity, semantic convergence, comprehension, clarity and difficulty of the items were evaluated. The scale was considered appropriate by content validation experts.

### Item analysis

The correlation between cases was 0.26 to 0.54. The highest correlation is related to case 14 and the lowest correlation is related to case 2. The results are presented in Table 1.

**Table 1:** Item-Total Statistics

	Corrected Item-Total Correlation	Cronbach’s Alpha if Item Deleted
CABLE-1.	.365	.884
CABLE-2.	.264	.886
CABLE-3.	.318	.885
CABLE-4.	.376	.884
CABLE-5.	.387	.884
CABLE-6	.447	.882
CABLE-7	.371	.884
CABLE-8.	.388	.883
CABLE-9	.536	.880
CABLE-10	.522	.880
CABLE-11.	.527	.881
CABLE-12.	.503	.881
CABLE-13.	.456	.882
CABLE-14.	.548	.880
CABLE-15.	.529	.880
CABLE-16.	.413	.883
CABLE-17.	.416	.883
CABLE-18.	.465	.882
CABLE-19.	.487	.881
CABLE-20.	.297	.886
CABLE-21.	.495	.881
CABLE-22	.526	.880
CABLE-23.	.432	.882
CABLE-24	.537	.880
CABLE-25.	.491	.881
CABLE-26	.362	.884
CABLE-27	.420	.883
CABLE-28.	.416	.883

α: Cronbach’s Alpha coefficient

### Construct validity

To ensure the suitability of the data for factor analysis, the KMO test was calculated to be 0.871. Bartlett test with respect to P < 0.001) (X^2^ was significant = 4511.146. In addition, PROMAX rotation method was used to extract factors and evaluate the validity of the structure. As a result, six factors − 28 Items with eigenvalues greater than 1 It was found that the cumulative variance explaining the concept by the instrument is 47.46% and acceptable. The results are presented in Table 2 and Fig. 1.

**Table 2:** Exploratory factor analysis of the farsi version of the CABLE

Factor	Items	Factor loading	h^2^	λ (% of Variance)	α	Ω	ICC
Help seeking	Q10. I took steps like creating future goals in order to rekindle the hope	.825	.305	2.850 (10.17)	0.894	0.896	0.848
	Q12. I tried to recreate and reshape myself through creating new goals/plans for my life	.794	.283				
	Q11. I tried to remind myself of the things that I am thankful for	.781	.382				
	Q9. I reminded myself of my positive attributes and potentials	.740	.452				
	Q8. I tried to actively focus on things I am doing to make progress, rather than being stuck on how difficult things are	.610	.479				
Positive outlook	Q19. I tried to seek comfort by revisiting my loved one’s belongings and keepsakes	.874	.369	2.870 (10.25)	0.822	0.836	0.894
	Q18. I talked to my loved one out loud or in my mind	.831	.295				
	Q18. I talked to my loved one out loud or in my mind	.831	.295				
	Q17. I looked at photos and or watched videos of my loved one	.790	.374				
	Q21. I revisited places that we used to go or did things that we used to do together	.671	.565				
	Q20. I kept a routine to be alone sometimes in order to express my grief and think about my loved one	.492	.673				
Spiritual support	Q5. I used professional resources (e.g., websites, grief literature, informational tools)	.701	.590	2.360 (8.42)	0.928	0.933	0.911
	Q4. I visited websites that provide help for those who have lost someone	.692	.628				
	Q3. I joined support groups that were specifically and methodically designed to help those who are in the process of bereavement	.623	.554				
	Q2. I attended grief therapy sessions provided by a mental health professional	.570	.392				
	Q1. I read self-help books to gain information about the grief process and coping mechanisms	.545	.461				
	Q6. I recorded positive changes and the progress I had made in the process of healing	.447	.609				
	Q7. I wrote helpful points and ways to overcome difficult situations on sticky notes and attached them in visible areas of the house as a reminder to use them during difficult times	.409	.693				
Continuing Bond	Q28. I tried to find people that could support me in my grief	.720	.737	1.795 (6.41)	0.870	0.876	
	Q25. I asked for the help and companionship of others	.698	.248				
	Q27. I tried starting new relationships or making new friends	.653	.524				
	Q26. I sought others’ positive feedback and approval	.594	.448				
Compassionate outreach	Q13. I took refuge in religion or spirituality (e.g., reading religious texts, prayer)	.716	.534	1.625 (5.80)	0.685	0.702	0.831
	Q16. I attended religious gatherings or services (e.g., in a mosque, church)	.687	.483				
	Q14. I relied on my spirituality to regain the sense of hope and inner peace	.671	.649				
	Q15. I talked to the higher power that I believe in about my grief	.421	.395				
Social support	Q23. I provided care or nurturance for others	.769	.433	1.796 (6.41)	0.764	0.773	0.792
	Q24. I engaged in benevolent and charitable activities	.580	.499				
	Q22. I told some close people how much I loved them and to what extent I cared for them	.501	.612				

**Abbreviations:** λ: Eigenvalue, h^2^: Communalities, α: Cronbach's alpha, Ω= McDonald Omega

**Fig. 2:**
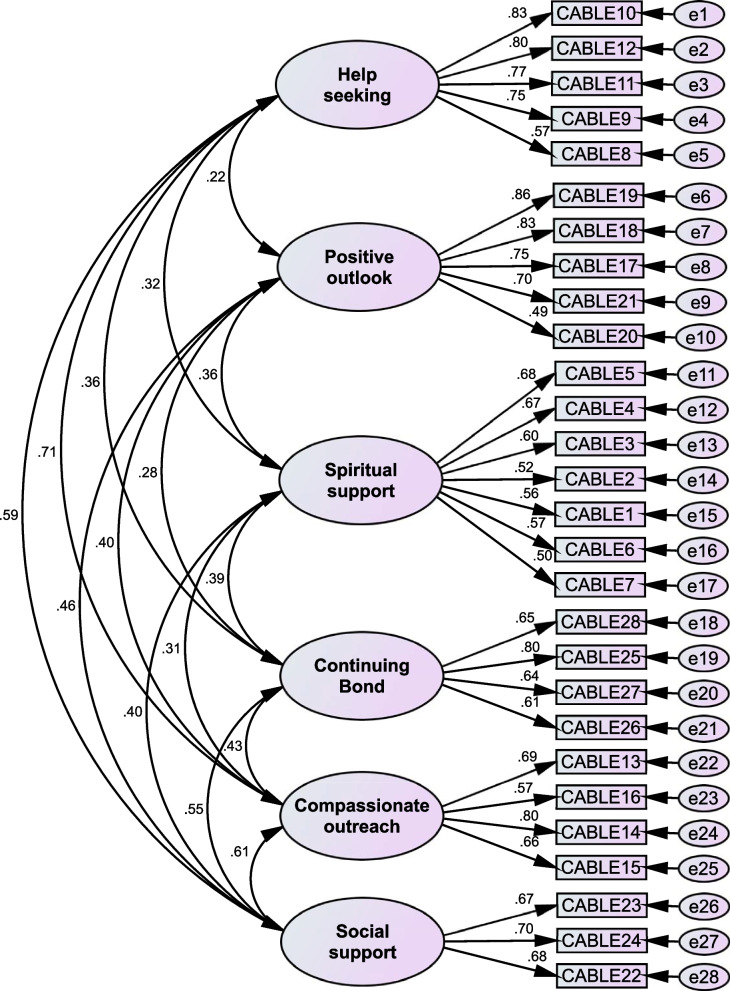
The final structure of the CABLE tool model

The results of confirmatory factor analysis also provided a good estimate based on the general fit indices of the model. The results are presented in Table 3. According to the final model of the factor structure of the CABLE structure, the variables showed a high correlation with their respective factor. The results are presented in Fig. 2.

**Table 3:** Values of fitting indices of CABLE tool confirmatory factor analysis pattern

Result	Fit Index
< 0.05	*P*-value 2_*X*_ (Chi-squared *P*-value)
335	Degrees of Freedom
800.68	Normal Theory Weighted Least Squares Chi-Square
0.058	RMSEA
2.37	CMIN/DF
0.92	NFI
0.96	CFI
0.88	GFI

### Reliability

In this study, the scale has an overall alpha coefficient of 0.910. Cronbach’s alpha and McDonald’s Omega were 0.894 and 0.820 for the first factor, 0.822 and 0.820 for the second factor, 0.928 and 0.70 for the third factor, 0.870 and 0.780 for the fourth factor, 0.685 and 0.770 for the fifth factor and 0.764 and 0.770 for the sixth factor. Also, in measuring the test stability by retesting method and using interclass correlation coefficient (ICC) with 95% confidence interval, the total ICC value is 0.862. ICC first factor is 0.848, second factor is 0.894, third factor is 0.911, fourth factor is 0.843, fifth factor is 0.831 and sixth factor is 0.792.

## Discussion

The aim of this study was to determine the psychometrics and validation of the CABLE scale (28 Items, 6 areas) in individuals living in Tehran. The designer of CABLE claims that this tool is the first tool designed to evaluate multidimensional, specific and cost-effective coping strategies that can be used in heterogeneous groups of mourners. This feature led to the selection of this tool to examine coping strategies among mourners, especially in the corona epidemic in Iran [25]. In the present study, the KMO value is equal to 0.871. This rate is reported to be 0.883 in the study of Crank et al., Who are the main designers of this tool. In the exploratory factor analysis, six factors (Help Seeking, Positive Outlook, Spiritual Support, Continuing Bonds, Compassionate Outreach, Social Support) were identified by the Promax rotation method, which corresponds to the number of factors identified in the study of Crank et al. [23, 25].

The eigenvalues in the study of Crank et al. [23, 25] were 8.4 and explained 57.81% of the variance, and the eigenvalues in the current study are 7.2, which explains 50% of the variance. To evaluate the internal consistency of the instrument, Cronbach’s alpha reliability coefficient was used, which is equal to 0.91, which is reported to be 0.89 in the study of Crank et al. [23, 25]. In both studies, the subsurface Compassionate Outreach factor is acceptable (in Crank et al.’s study equal to 0.66 and in the current study 0.68 but due to the fulfillment of all pre-determined psychometric criteria in each two studies have been preserved. In the study of Crank et al. [23, 25]. The correlation between the items in the correlation matrix is from 0.00 to − 0.3 and in the current study it is from 0.4 to 0.8. A review of the literature shows that the minimum acceptable value for the correlation between items is 0.15. Therefore; The correlation of all items is appropriate. In the study of Crank et al.; The results of confirmatory factor analysis of the items are as follows: RMSEA = 048/0 CFI = 924/0 NFI = 81/0 RMR = 078/0 And these indicators in the current study are as follows: RMSEA = 058/0 CFI = 96/0 NFI = 92/0 0 RMR = 056/0 As can be seen in the literature review, CABLE was first designed and psychoanalyzed in 2017 by Crank et al. [23, 24] and the results of the current study are somewhat similar to the results and findings of the original designers and it seems that in different cultures and religions, adapting and coping with stress is a condition for survival. There are various types of mourning for loved ones that based on contemporary models of adaptation to mourning (dual process model and meaning reconstruction model), people without the choice between two ends of a continuum to look for coping strategies to alleviate grief and accept new conditions. There are many similarities between adults with the experience of grief in different cultures, which may be related to the globalization of culture.

## Conclusion

The findings of the present study show that the CABLE scale in Iran has good validity and reliability and can be used to evaluate the coping strategies of adults in mourning for the purposes of psychological research, sociology or cultural policy. Based on the findings, CABLE has good credibility and reliability in Iran and can be used to evaluate the coping strategies of adults in mourning for the purposes of psychological research, sociology or cultural policy.

## Limitations

This study was performed on adults living in Tehran and often electronically. It is suggested that this study be performed in person in adults living in other cities with cultural diversity and in person.

## Data Availability

The datasets generated and/or analyzed during the current study are available from first and Corresponding author of this article upon reasonable request.
